# Differences in Copper Absorption and Accumulation between Copper-Exclusion and Copper-Enrichment Plants: A Comparison of Structure and Physiological Responses

**DOI:** 10.1371/journal.pone.0133424

**Published:** 2015-07-24

**Authors:** Lei Fu, Chen Chen, Bin Wang, Xishi Zhou, Shuhuan Li, Pan Guo, Zhenguo Shen, Guiping Wang, Yahua Chen

**Affiliations:** College of Life Sciences, Jiangsu Collaborative Innovation Center for Solid Organic Waste Resource, National Joint Local Engineering Research Center for Rural Land Resources Use and Consolidation, Nanjing Agricultural University, Nanjing, Jiangsu, China; Henan Agricultural Univerisity, CHINA

## Abstract

Differences in copper (Cu) absorption and transport, physiological responses and structural characteristics between two types of Cu-resistant plants, *Oenothera glazioviana* (Cu-exclusion type) and *Elsholtzia haichowensis* (Cu-enrichment type), were investigated in the present study. The results indicated the following: (1) After 50 μM Cu treatment, the Cu ratio in the xylem vessels of *E*. *haichowensis* increased by 60%. A Cu adsorption experiment indicated that *O*. *glazioviana* exhibited greater resistance to Cu, and Cu absorption and the shoot/root ratio of Cu were significantly lower in *O*. *glazioviana* than in *E*. *haichowensis*. (2) An analysis of the endogenous abscisic acid (ABA) variance and exogenous ABA treatment demonstrated that the ABA levels of both plants did not differ; exogenous ABA treatment clearly reduced Cu accumulation in both plants. (3) The leaf stomatal density of *O*. *glazioviana* was significantly less than that of *E*. *haichowensis*. Guard cells in *E*. *haichowensis* plants were covered with a thick cuticle layer, the epidermal hair was more numerous and longer, and the number of xylem conduits in the root was small. (4) The transpiration rate and the stomatal conductance of *O*. *glazioviana* were both significantly lower than those of *E*. *haichowensis*, regardless of whether the plants were treated with Cu. Taken together, these results indicate that the differences in the structural characteristics between these two plant species, particularly in the characteristics related to plant transpiration, are important factors that govern whether plants acquire or exclude Cu.

## Introduction

Heavy-metal pollution in soil has become a serious environmental problem in recent decades. Heavy metals comprise an ill-defined group of inorganic chemical hazards, and the heavy metals that are most commonly found at contaminated sites are lead (Pb), chromium (Cr), arsenic (As), zinc (Zn), cadmium (Cd), copper (Cu), mercury (Hg), and nickel (Ni) [1]. Heavy-metal soil contamination may pose risks to humans and to ecosystems through the direct ingestion or contact with contaminated soil, contamination of the food chain (soil-plant-human or soil-plant-animal-human), the consumption of contaminated ground water, a reduction in food quality (safety and marketability) via phytotoxicity, a reduction in land usability for agricultural production that results in food insecurity, and land tenure problems [2].

Plants have developed complex mechanisms for transporting mineral nutrients to optimise their growth and reproductive success over a range of environmental conditions [3]. In general, metal ions are removed from the soil via a low-resistance apoplastic or symplastic pathway and transported to other parts of the plant through the xylem[4,5]. At the whole-plant level, long-distance transport is essential for the distribution of a variety of mineral nutrients [6]. Transpiration, which is the evaporative loss of water from plant leaves, is believed to be one of the most important components of the long-distance transport of inorganic nutrients in the xylem of higher plants [7]. In some model plants, heavy-metal toxicity has been shown to be dependent on several physiological and biochemical mechanisms, among which a low transpiration rate (Tr) may play an important role in excluding heavy metals [8]. Transpirational pull has been demonstrated to be similarly important in the process of Se accumulation and translocation in wheat and spring canola [9].

*Elsholtzia haichowensis*, which is a representative Cu-resistant plant, can accumulate large numbers of Cu ions, and its physiological responses to copper exposure [10,11], ability to absorb copper [12], resistance to copper [13], proteomic characterization under copper stress[14], and ability to phytoremediate copper-contaminated soils have been intensively studied [15,16]. *Oenothera glazioviana* is a member of the evening primrose plant family (Onagraceae, Myrtales, *Oenothera* L.). We previously showed that *O*. *glazioviana*, which is a Cu excluder and an edible seed crop of high economic value, is a dominant species in semiarid mine tailings and exhibits low Cu translocation and accumulation[17]. Thus, *O*. *glazioviana* is an environmentally friendly plant that is used to promote stable plant technology in areas with Cu tailings. The term excluder plant was first documented in 1981 by Baker[18], but studies on this field are still limited[19]. Under certain conditions, *Oenothera biennis* can simultaneously exclude the uptake of Cd and Cu, to screen excluder plants based on weed species could make agreat breakthrough in phytostabilization and safe agroproduction for soils contaminated by heavy metals [20]. It was evaluated that the interactive effect between sugar beet (SB) amendment and Cu-adapted AM fungi could be a successful biotechnological tool for improving the metallophyte *Oenothera piscensis* establishment in highly Cu-polluted soils[21,22]. Copper extraction by Oenothera picensis in Cu-contaminated acid soils was increased 14 times by the addition of 6 to 10 mmol plant^-1^ of methylglycinediacetic acid [23]. However, the mechanisms responsible for low copper accumulation in *O*. *glazioviana* remain unclear. In this study, hydroponic, leaf-epidermis and cell-wall Cu accumulation analyses were conducted to investigate the differences between *E*. *haichowensis* and *O*. *glazioviana*, particularly in terms of the characteristics of their tissue structures, to study the mechanisms of Cu resistance in greater depth.

## Materials and Methods

### Ethics statement

The seeds of *O*. *glazioviana* were collected fromYangshanchong copper mine tailings (30°54′N, 117°53′E) in the city of Tongling in Anhui Province, China. *E*. *haichowensis* seeds were collected from Tangshan (32°03′N, 118°547′E) in the city of Nanjing in Jiangsu Province, China. Both the two species are not endangered or protected species in China. As the locations are open to the public, no specific permissions were required for these activities.

### Plant materials and growth conditions

Uniform and healthy seeds of *O*. *glazioviana* and *E*. *haichowensis* were chosen, soaked in tap water for 24 h and then sown in plastic pots filled with vermiculite. The seeds were germinated and grown in a growth chamber with a light cycle of 12 h light/12 h dark and day/night temperatures of 25°C/20°C. The cotyledons of each cultivar fully opened after approximately 15 days. The seedlings were fixed in cystose and transferred to a vessel containing 2 L of 0.5× Hoagland’s nutrient solution at a pH of 5.6 ± 0.1. The Hoagland nutrient solution consisted of 5 mM Ca(NO_3_)_2_, 5 mM KNO_3_, 1 mM KH_2_PO_4_, 50μM H_3_BO_3_, 1 mM MgSO_4_, 4.5 μM MnCl_2_, 3.8 μM ZnSO_4_, 0.32 μM CuSO_4_, 0.1 mM (NH_4_)_6_Mo_7_O_24_ and 10 μM Fe EDTA. Three replicates with three plants in experiments as following were conducted, and the nutrient solutions were renewed every 3 days.

### Features of Cu accumulation in two plant species

The 14-day-old seedlings were treated with 50 μM Cu for 1 h, 3 h, 6 h, 10 h, 24 h, 72 h, 192 h, and 384 h. Each treatment was replicated in three different vessels, with each vessel containing five plants. The plants were harvested, and the roots were soaked in 25 mM EDTA-Na solution for 15 min to desorb the metal ions on the surfaces of the roots. Then, the leaves and roots were separated, washed thoroughly with tap water, rinsed with deionised water, blotted dry with tissue paper, exposed to a temperature of 120°C for 0.5 h to deactivate enzymes, and dried at 80°C for 24 h. The weights and Cu contents of the leaves and roots were then determined, and the transfer coefficient was calculated using the following equation: translocation factor (TF) = copper content in shoot/copper content in root [24].

### Cu distribution in root cross sections

Cu distribution analysis was performed on root cross sections using the method described by Isaure et al.[25]. The roots were treated with 50 μM Cu for 3 days and then washed with deionised water. Next, 3.0–3.5-cm long root apices were quickly frozen in liquid nitrogen (-196°C), freeze-dried at -50°C for 10 h, and then dried in a vacuum for 24 h. The samples were observed using a scanning electron microscope (SEM; XL30 ESEM, Philips Co., Netherlands) after being sprayed with a gold film. The weight of each element as a percentage of the total weight of each root cross section was analysed using an energy-dispersive spectrometer (EDS, Kevex, Noran Co., USA).

An SEM X-ray energy spectrum analyser was used to determine which elements were present in the root cross sections. A small area of each sample was bombarded by an electron beam, the resulting electron transitions generated X-rays, and the characteristic X-ray-energy spectrum of every element was then displayed on the screen of the SEM X-ray energy spectrum analyser. The horizontal position of the spectral line indicates the energy of these X-rays, and the height of the spectral line represents their intensity. Finally, the relative weight of each element was calculated as a percentage, using a non-standard sample method (Wt %).

### Cu accumulation and adsorption in plant cell walls

#### Cu accumulation in complete cell walls

Next, the Cu content that had accumulated in complete cell walls was analysed. Complete root samples from the two species of plants were treated with 50 μM CuSO_4_ for 3 days, harvested, and washed with deionised water. Root cell walls were extracted using the method described previously by Hart et al.[26]. The root samples were soaked in methanol:chloroform (MC) solution (methanol:chloroform = 1:2) for 3 days, rinsed thoroughly and dried at 60°C. The Cu content was determined compared with those of roots that had not been treated with MC, as the controls.

#### Cu adsorption in crude cell walls extractions

Crude cell wall extractions were performed using the method described previously by Zhong and Läuchli and by Zheng et al. [27,28]. Frozen plant samples were ground using a mortar in the presence of liquid nitrogen. The resulting powdered plant samples were extracted using three 15-ml volumes of chilled 75% ethanol. The mixture was transferred to a 50-ml centrifuge tube, stirred, allowed to stand for 20 min, and centrifuged (4000×g) for 10 min at 4°C. The resulting pellet was washed sequentially with cold acetone (root weight (g): volume of acetone (ml) = 1:7), a methanol:chloroform mixture (v:v = l:l) and a methanol solution. After every wash, the suspension was centrifuged (5000 × g) for 10 min at 4°C. The precipitate was freeze-dried (Christ Alpha l-4, Germany), ground in liquid nitrogen, and stored at 4°C until use.

Adsorption experiments were performed using the method described previously by Wu et al. with slight modifications [16]. The adsorption solution was 50 μM CuSO_4_, and 0.01 M KNO_3_ was used as the electrolyte solution (pH 5.7, 25°C). First, 0.05 g of a cell wall sample was carefully weighed and placed in a homemade filter with filter cloth at the bottom. Both ends of the filter had connectors to connect to the adsorption solution and to collect the effluent. The adsorption solution was pumped into the collection tube at a rate of 3 ml·10 min^–1^ using a peristaltic pump and then collected using an automatic collector after flowing through the cell wall sample; one tube was collected every 10 min. The collection was terminated when the Cu concentration in the effluent was identical to that in the absorption solution and reached the adsorption balance (approximately 900 min). The Cu content in each tube was then determined by flame atomic absorption (AAS).

### Effect of Cu on plant transpiration

Fourteen-day-old seedlings were treated with 50 μM Cu for 3 days, and control seedlings underwent the same treatment as treated seedlings except that Cu was not present in the liquid with which the control seedlings were treated. The leaves of the two species of plants were used to determine the Tr, the stomatic conductance (Gs), and the intercellular CO_2_ concentration (Ci), using a portable photosynthesis system (Li-6400, Li-Cor Inc., USA).

### Correlation between the Tr and Cu accumulation in plants

First, 14-day-old seedlings exhibiting similar growth and of similar vigour and size were fixed in completely sealed 25-ml tubes, and 20 ml of 50 μM CuSO_4_ solution (prepared using Hoagland’s nutrient solution) and several concentrations of PEG (polyethylene glycol 4000) were then accurately added to the tubes. The following concentrations of PEG, in the presence of 50 μM Cu, were added to the tubes: 0, 0.005%, 0.01%, 0.03%, 0.05%, 0.075%, 0.1%, 0.2%, 0.3%, 0.4%, 0.5%, 0.6%, 0.7%, 0.8%, 0.9%, 1%, 2%, 3%, 4%, 5%, 6%, 7%, 8%, 9%, and 10% (n = 25). After five days of treatment, the volume (V, ml) of the remaining liquid in each tube was recorded, the weight (W, g) of the plant leaves corresponding to each tube was measured, and the Cu contents in the roots and the shoots were determined. The Tr was then calculated as follows[29]:

$$\text{Tr}=\frac{\left(20-\text{V}\right)}{3\text{W}}(\;\text{mg}/[\;\text{g}\cdot \text{d}]\;)$$

### Effect of Cu on the abscisic acid (ABA) content in plants

Fourteen-day-old seedlings were treated with 50 μM Cu for 3 days, and seedlings not treated with Cu were used as controls. Then, the roots and leaves of the seedlings were harvested for ABA content analysis using enzyme-linked immunosorbent assays (ELISAs; ADL Co., USA)[30].

### Effect of exogenous ABA on the Tr and the Cu content in plants

The 14-day-old seedling treatment groups were as follows: 10 μM ABA, 50 μM Cu, 10 μM ABA+50 μM Cu, and the control group. The control group were not treated with ABA or Cu. The plants were harvested after 3 days to analyse the Tr and Cu concentrations in the roots and shoots of the seedlings.

### Analysis of the wax content of plant leaves

To determine the relationship between leaf structure and copper tolerance in the two plant species examined in our study, leaves of each plant species were carefully harvested, and the wax contents of the leaves were measured. The total wax content of leaf samples was determined by chloroform extraction [31].

### Structural analysis of the leaf hypodermis

To view the plant leaf hypodermis by SEM, leaf samples were cut into 1-cm^2^ pieces and fixed in formalin–acetic acid–alcohol (FAA) for 48 h, repeatedly dehydrated with ethanol (70%, 85%, 95%, and 100%) and fixed on a work table with double-sided adhesive. The samples were then subjected to CO_2_ critical point drying, sprayed with gold and imaged using a Philips XL30 ESEM operating at 30 kV [32]. Finally, ten images of each sample were randomly selected, and the structure of the hypodermis was analysed.

### Microstructural analysis of root cross sections

To observe the microstructure of plant tissue, root paraffin section were prepared, and electron micrographs of plant regions were acquired. Briefly, 3-5-mm slices of fresh roots adjacent to the cut edge were cut into 0.5-cm^2^ pieces and fixed in FAA for 48 h. Permanent paraffin sections were prepared in a series of steps that included washing, dehydrating, rendering transparent, paraffin-embedding, sealing, slicing, dewaxing, dyeing, dehydrating, rendering transparent, sealing, and labelling samples with the production date. Then, the samples were observed and imaged using an optical microscope (Carl Zeiss, Jena, Germany). Another part of the same root system was pretreated using the methods described above, plated with gold film and observed under an SEM (XL30 ESEM, Philips Co., Netherlands).

### Plant analysis

The dried plant tissues were weighed and powdered for Cu concentration analysis. The powdered plant material was wet digested in heat-resistant glass tubes in a heating block with an 87:13 (v:v) mixture of nitric acid and perchloric acid [33]. The digests were eventually dissolved in 5% HNO_3_ for Cu analyses using a NOVA 300 atomic absorption spectrophotometer (Analytik, Jena, Germany).

### Statistical analysis

Single-factor analyses of variance were performed, after which Duncan's multiple range test was used. A significance level of *p*<0.05 was used for all analyses. SPSS software (ver. 14.0; SPSS Inc., Chicago, IL, USA) was used for the statistical analyses.

## Results

### Cu uptake, transport and distribution in *O*. *glazioviana* and *E*. *haichowensis*

#### Dynamic accumulation of Cu in *O*. *glazioviana* and *E*. *haichowensis* shoots and roots

**Fig 1:**
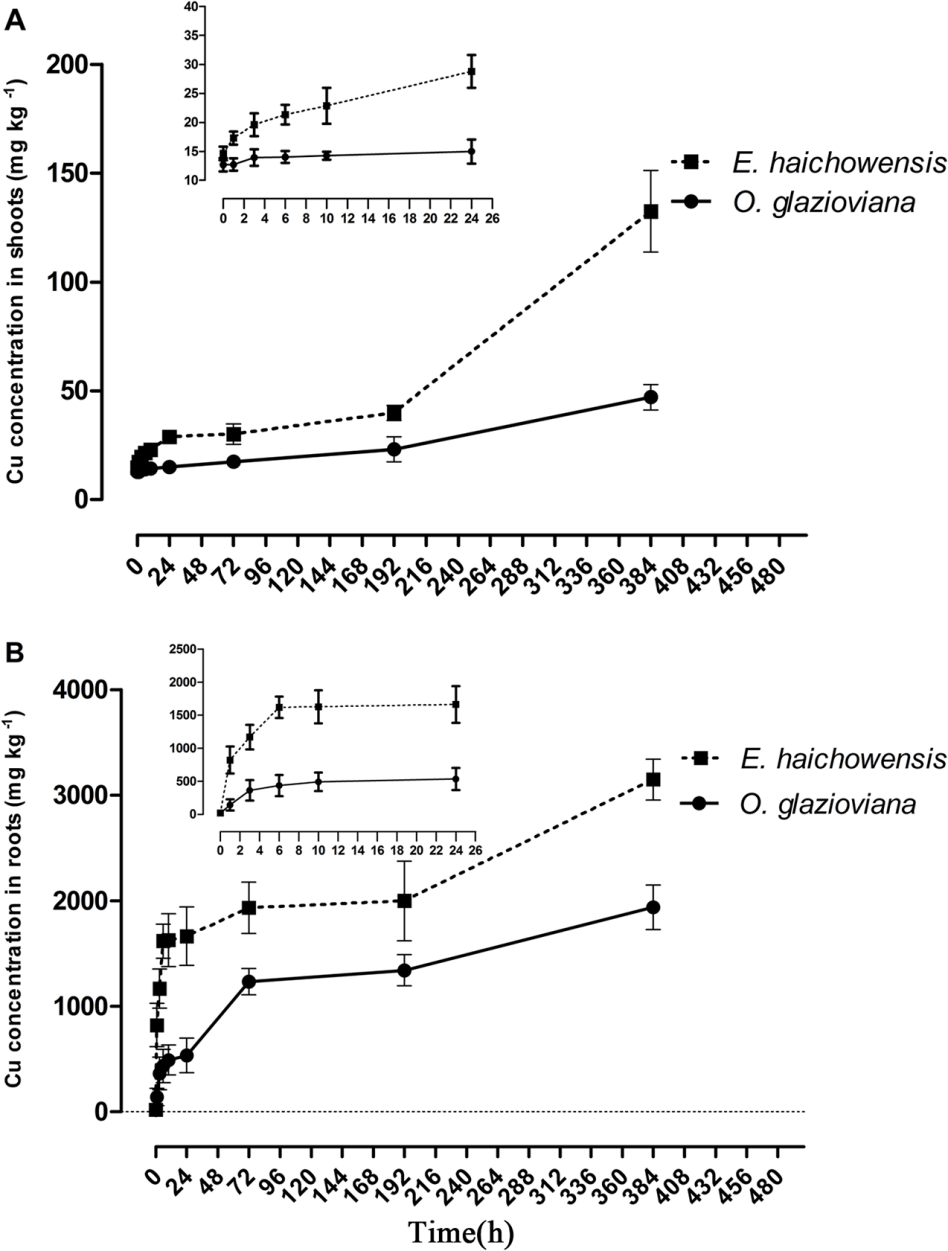
Dynamic Cu accumulation in the shoots (A) and roots (B) of *Oenothera glazioviana* and *Elsholtzia haichowensis* seedlings after 50 μM Cu treatment. Mean value followed by different letter is statistically significant (ANOVA; Duncan multiple range test, p<0.05).

*O*. *glazioviana* and *E*. *haichowensis* plants that were treated with 50 μM Cu were used to examine the dynamic processes of Cu uptake in roots and Cu transport to shoots. As shown in Fig 1, the root Cu content of both plants increased gradually as the treatment time increased. After 1 h of Cu exposure, the Cu content in the roots of both plants presented significant differences. Cu accumulation in *E*. *haichowensis* roots reached 822 mg kg^-1^ dry weight (DW), which was significantly higher than that of *O*. *glazioviana* during the entire processing time.

The Cu transport to the shoots of both plants was also very different between the two species. When exposed to Cu for 1 h, the Cu content in the shoots of *E*. *haichowensis* exhibited a significant upward trend; however, the Cu content in the shoots of *O*. *glazioviana* exhibited no obvious change within 24 h. After 192 h, the Cu contents in the shoots of *E*. *haichowensis* and *O*. *glazioviana* were 1.62 and 3.07 times those of the corresponding controls, respectively. After 384 h, Cu accumulation in the shoots of *E*. *haichowensis* increased to 132 mg kg^-1^ DW, whereas that in the shoots of *O*. *glazioviana* reached only 47.1 mg kg^-1^ DW. These results demonstrated that Cu translocated more rapidly and in greater quantities into the shoots of *E*. *haichowensis* than into those of *O*. *glazioviana*.

#### Cu content in xylem vessels and in the cell walls in root tips

Cu accumulation in the cell walls and xylem vessels were determined by X-ray energy spectral analysis. The analysis was conducted in the root tips of both plants, which were treated with 50 μM Cu for three days (Table 1). Notably, the percentages of Cu accumulation in the cell walls increased by 25.4% and 29.8% in *O*. *glazioviana* and *E*. *haichowensis*, respectively, compared with the controls. Cu accumulation in the cell walls of *E*. *haichowensis* was 1796 mg kg^-1^ DW, which was significantly higher than that of *O*. *glazioviana* (1108 mg kg^-1^ DW). The Cu ratio in the root xylem vessels of *O*. *glazioviana* treated with Cu increased from 17.9 Wt% to 19.6 Wt% (increased 0.09 times); however, the Cu ratio increased from 34.1 Wt% to 54.3 Wt% in *E*. *haichowensis* (increased by a factor of 1.6), which was higher than the observed increase in the Cu ratio in *O*. *glazioviana*.

**Table 1:** Cu contents in xylem vessels and cell walls in the roots of *O*. *glazioviana* and *E*. *haichowensis* seedlings after 0 or 50 μM Cu treatment for 3 days.

Species	Cu content in vessel (Wt %)		Cu content in cell wall (Wt %)	
	Control	50 μM	Control	50 μM
*O*. *glazioviana*	17.91±0.83	19.61±1.03	72.51±3.13	90.92±4.15
*E*. *haichowensis*	34.05±1.75	54.33±2.44	73.68±3.57	95.64±4.69

#### Cu adsorption rate in root cell walls

**Fig 2:**
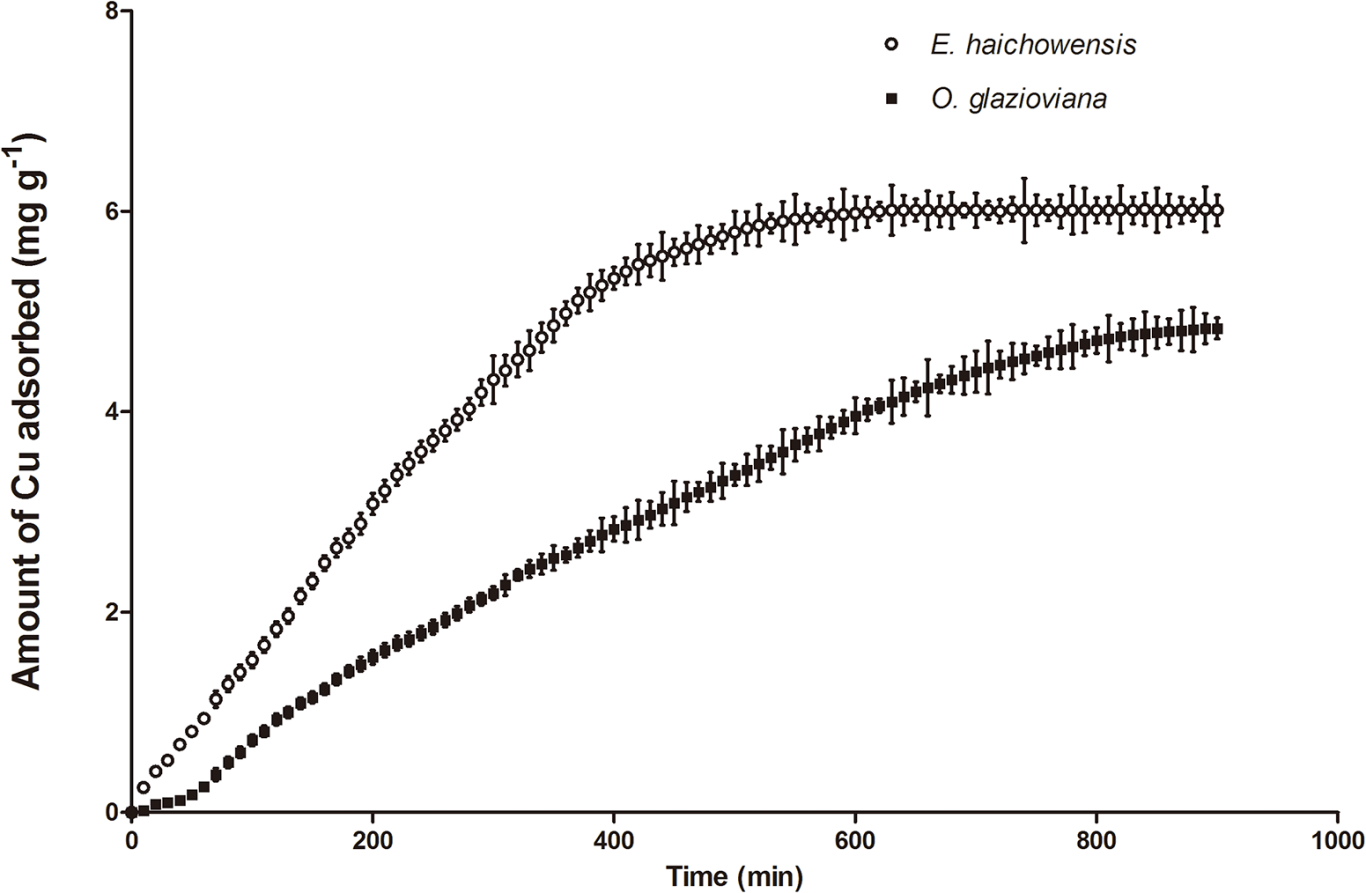
The adsorption of Cu^2+^ in the root cell walls of *O*. *glazioviana* and *E*. *haichowensis*. Mean value followed by different letter is statistically significant (ANOVA; Duncan multiple range test, p<0.05).

Fig 2 shows the dynamic changes in cell wall Cu^2+^ adsorption in both plants (25°C, pH 5.7). During the initial stage, the Cu^2+^ adsorption rates of the root cell walls in both plants were fast. As time increased, the changes in the adsorbed quantities became increasingly smaller. Then, by 600 min, *E*. *haichowensis* root cell walls gradually reached adsorption equilibrium; however, *O*. *glazioviana* root cell walls reached adsorption equilibrium at 900 min. The maximum Cu^2+^ adsorption quantity of *E*. *haichowensis* root cell walls was 5.98 mg g^-1^, which was significantly higher than that of *O*. *glazioviana* cell walls, at 4.31 mg g^–1^. These results further demonstrate that *O*. *glazioviana* maintains its high resistance to Cu by limiting Cu accumulation and Cu adsorption in cell walls.

### The correlation between plant transpiration and the Cu transfer process

The leaf stomatal conductance and Tr of *O*. *glazioviana* were approximately 1/3 those of *E*. *haichowensis* when both plants were treated without additional Cu. However, the Ci of *O*. *glazioviana* was slightly lower than that of *E*. *haichowensis* (Table 2). Compared to the controls, with 50 μM Cu treatment, the Tr of *E*. *haichowensis* decreased by 34.86% to 7.24 mmol H_2_O m^-2^ s^-1^ but was higher than that of *O*. *glazioviana*, which was 3.20 mmol H_2_O m^-2^ s^-1^ under the same conditions.

**Table 2:** Effects of Cu treatment on the transpiration rate, intercellular CO_2_ concentration, and stomatal conductance in leaves of *O*. *glazioviana* and *E*. *haichowensis* seedlings.

Plant species	Treatment (μM)	Transpiration rate (mmol H_2_O m^-2^ s^-1^)	Stomatal conductance (mol H_2_O m^-2^ s^-1^)	Intercellular CO_2_ concentration (μmol CO_2_ mol^-1^)
*O*. *glazioviana*	0	4.28±0.43 c	0.285±0.063 b	289±19 bc
	50	3.20±0.30 d	0.164±0.004 c	259±19 c
*E*. *haichowensis*	0	11.1±0.85 a	0.715±0.157 a	322±5 a
	50	7.24±1.40 b	0.325±0.046 b	299±12 b

Mean value followed by different letter is statistically significant (ANOVA; Duncan multiple range test, p<0.05).

**Fig 3:**
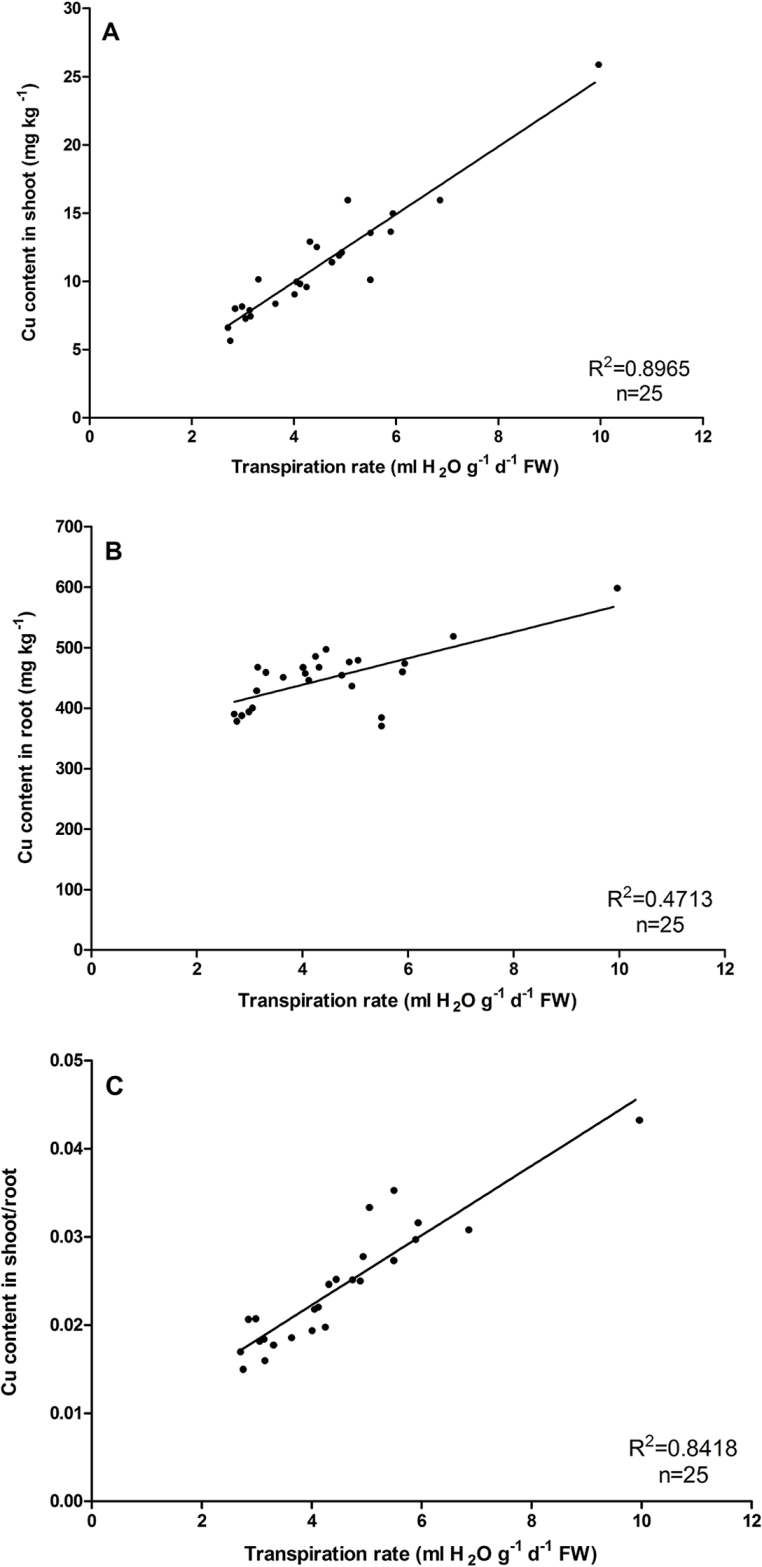
Relationships between the shoot Cu concentration (A), root Cu concentration (B) and the ratio of the Cu concentration in shoots to that in roots of *O*. *glazioviana* (C) and the leaf transpiration rate. Mean value followed by different letter is statistically significant (ANOVA; Duncan multiple range test, p<0.01).

**Fig 4:**
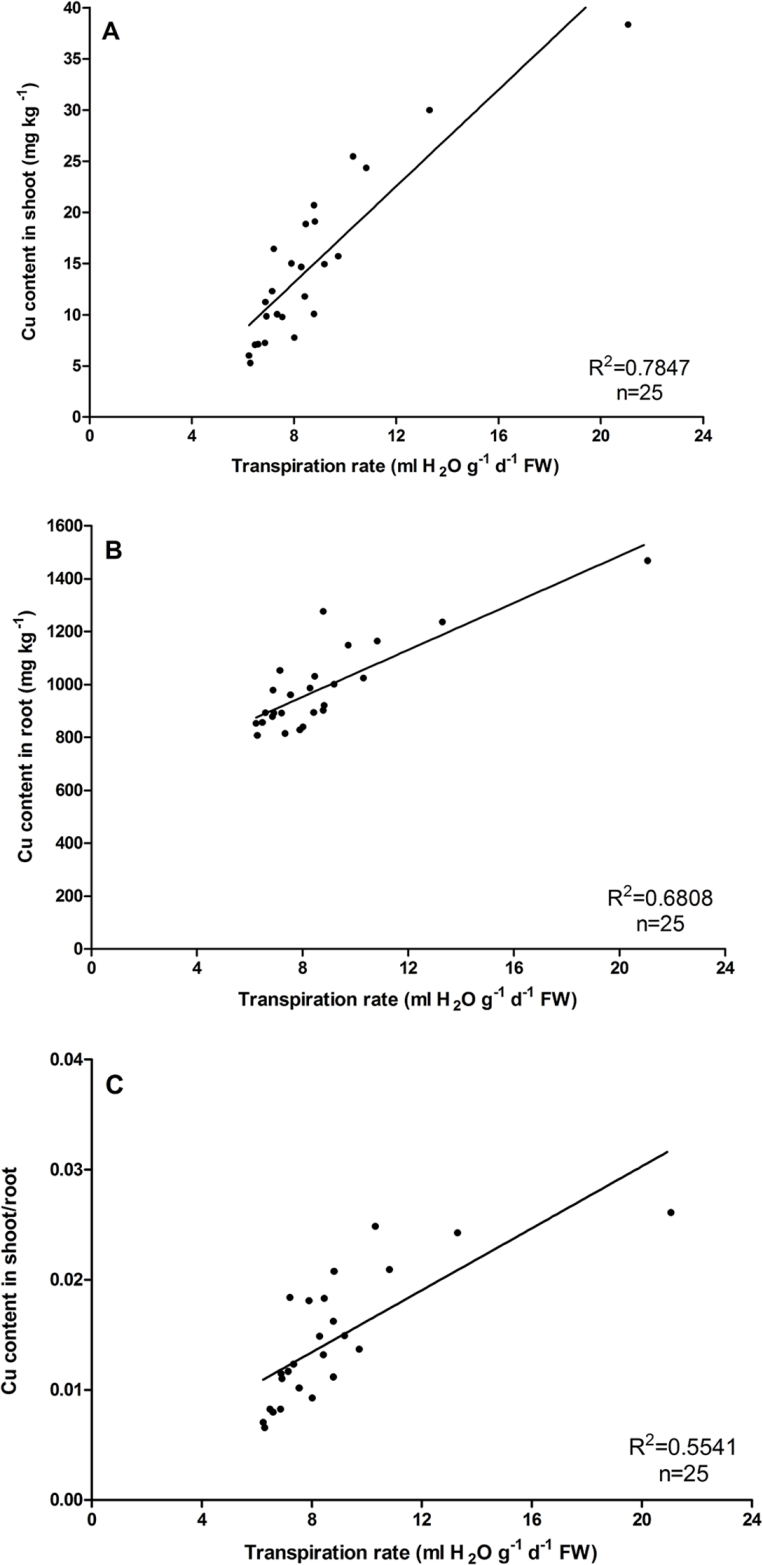
Relationships between the shoot Cu concentration (A), root Cu concentration (B) and the ratio of the Cu concentration in shoots to that in roots of *E*. *haichowensis* (C) and the leaf transpiration rate. Mean value followed by different letter is statistically significant (ANOVA; Duncan multiple range test, p<0.01).

The relation between the Cu transfer process and the Tr in *O*. *glazioviana* and *E*. *haichowensis* was studied using different PEG concentrations to stimulate drought conditions, result in limiting transpiration. Fig 3 shows a significant positive correlation between the shoot and root Cu contents of *O*. *glazioviana* and the Tr (R^2^ = 0.8976 and R^2^ = 0.4713, n = 25), while the Cu transfer coefficient also positively correlated with the Tr (R^2^ = 0.8418, n = 25). As in Fig 4, the Cu concentrations in *E*. *haichowensis* shoots and roots also increased significantly with increasing leaf transpiration (R^2^ = 0.7848 and R^2^ = 0.6808). A significant increase in the shoot/root ratio of Cu concentrations was occurred with increasing leaf transpiration (R^2^ = 0.5541).

On the other hand, the treatment of PEG had more significant effect on reducing the shoot and root Cu contents in *E*. *haichowensis* compared with *O*. *glazioviana*. The content of Cu concentration in shoot and root decreased 32.32mg kg^-1^ and 615.23mg kg^-1^ in *E*. *haichowensis*, the corresponding decreases in *O*. *glazioviana* were only 19.26mg kg^-1^ and 208.30mg kg^-1^.All these results indicate the Tr could significantly affect the Cu transfer process both in *O*. *glazioviana* and *E*. *haichowensis*. The low Tr of *O*. *glazioviana* in natural conditions may be the primary reason for the lower Cu accumulation, in contrast with a higher Tr in *E*. *haichowensis*.

### Changes in transpiration and Cu content induced by endogenous ABA and exogenous ABA treatments

**Fig 5:**
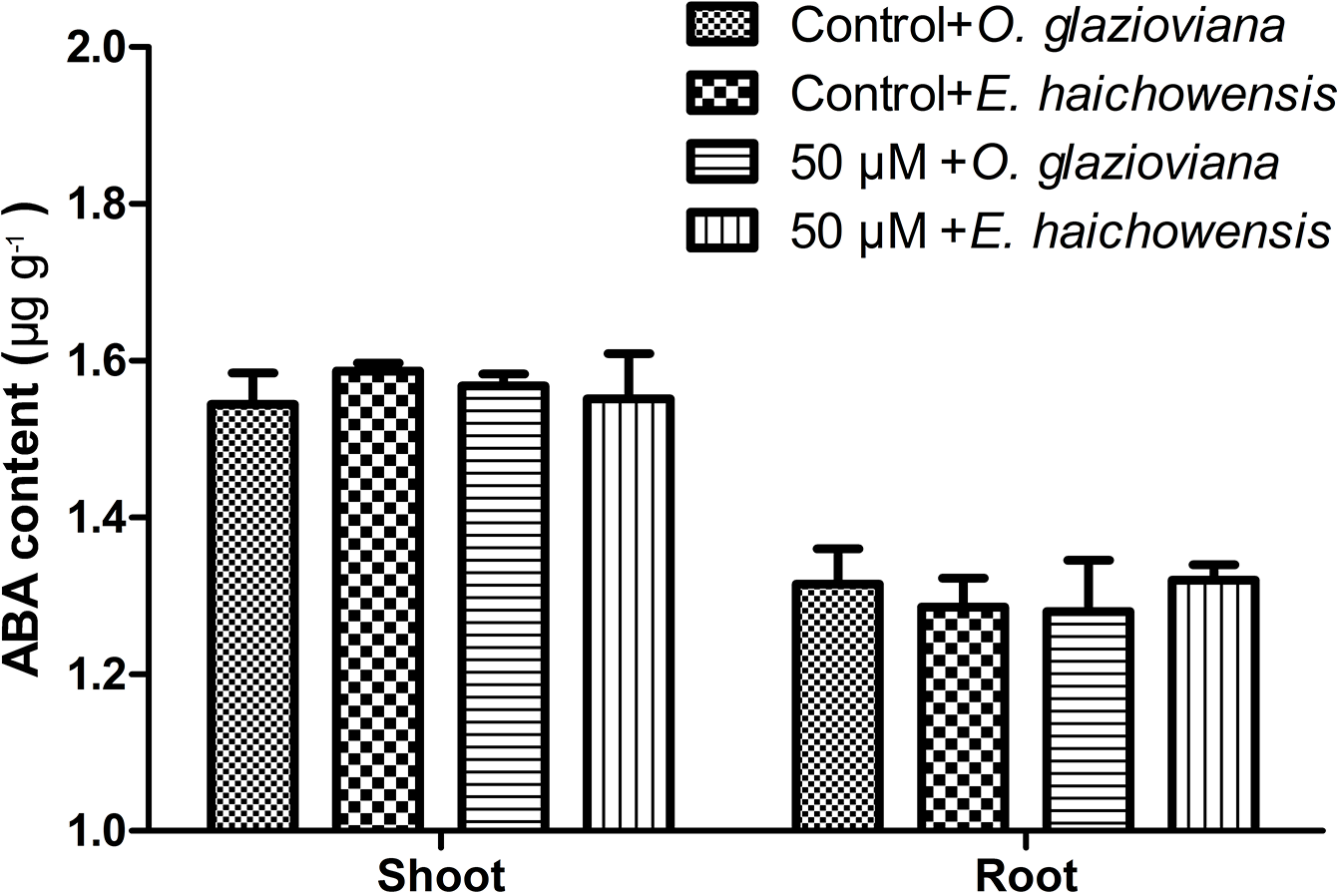
ABA contents in the leaves of *O*. *glazioviana* and *E*. *haichowensis* seedlings with treatment with 0 or 50 μM CuSO_4_. Mean value followed by different letter is statistically significant (ANOVA; Duncan multiple range test, p<0.05).

To study the key factors that influence *O*. *glazioviana* transpiration, ELISAs were used to detect the ABA content in both plants (Fig 5). The ABA levels in these two types of plants did not differ under normal conditions or with Cu treatment. The ABA contents of the shoots were higher compared with those of the roots in both plants, and Cu treatment did not change the ABA levels.

As shown in Table 3, the Tr in *O*. *glazioviana* was significantly inhibited by 10 μM ABA and by 50 μM Cu. The Cu content in *O*. *glazioviana* shoots did not change significantly in the absence of ABA after 50 μM Cu was added to the nutrient solution, while the root Cu content increased by a factor of 53.4. In the absence of Cu, exogenous ABA significantly decreased the Cu content in both the shoots and roots of *O*. *glazioviana*, and ABA eased Cu uptake in roots when a solution containing additional ABA and Cu was added.

**Table 3:** Effects of ABA pretreatment (10 μM) on Cu contents in the shoots and roots of *O*. *glazioviana* and *E*. *haichowensis* seedlings treated with 0 or 50 μM CuSO_4_.

	ABA treatment (10 μM)	Cu^2+^ treatment (50 μM)	Transpiration rate (ml H_2_O g^-1^ d^-1^ FW)	Cu in Shoots (mg kg-1)	Cu in Roots (mg kg-1)
*O*. *glazioviana*	−	−	10.7±0.5	10.7±1.7	18.5±1.8
	−	+	8.3±0.5	12.2±1.2	919.2±46.5
	+	−	5.3±0.6	6.7±1.6	11.9±1.5
	+	+	4.9±0.4	9.0±1.7	640.5±202.3
	*ANOVA F ratio*				
	Cu		22.48**	3.83 ^NS^	212.54**
	ABA		221.78**	13.95**	8.03*
	Cu×ABA		11.43*	0.21 ^NS^	7.33*
	Error df		6	6	6
*E*. *haichowensis*	−	−	27.8±3.7	12.7±1.5	19.3±3.2
	−	+	20.6±1.5	24.9±2.8	1248.4±129.6
	+	−	11.6±1.7	5.9±1.7	12.4±2.3
	+	+	11.1±1.7	7.1±1.8	1163.0±57.5
	*ANOVA F ratio*				
	Cu		6.75*	31.20**	682.33**
	ABA		74.98**	105.59**	1.03^NS^
	Cu×ABA		5.22 ^NS^	21.52**	0.74^NS^
	Error df		6	6	6

NS: not significant

**P<0.01

*P<0.05.

The Tr of *E*. *haichowensis* was also significantly inhibited by 10 μM ABA and 50 μM Cu (Table 3). In the absence of ABA, 50 μM Cu significantly promoted a 96% increase in the Cu content of *E*. *haichowensis* shoots, and the Cu content in *E*. *haichowensis* roots increased by 86.7%. Similar to *O*. *glazioviana*, Cu accumulation in *E*. *haichowensis* shoots was more significantly affected by exogenous ABA; exogenous ABA reduced Cu accumulation in shoots by 71.49%. When plants were treated with a solution containing both ABA and Cu, exogenous ABA significantly decreased the Cu content in *E*. *haichowensis* shoots but not in the roots.

### Structural analysis of plant leaves

#### Stomatal density and wax content in the plant hypodermis

Under normal conditions, the stomatal densities in the reciprocal second and third leaves of *O*. *glazioviana* were significantly lower than those in *E*. *haichowensis*. However, the wax contents of these leaves were significantly higher than the wax content of *E*. *haichowensis* leaves (Table 4).

**Table 4:** Wax content in leaves and stomatal density of the leaf epidermis of *O*. *glazioviana* and *E*. *haichowensis* seedlings.

Species	Wax content (mg g^-1^)	Stomatal densities in top second leaf (mm^-2^)	Stomatal densities in top third leaf (mm^-2^)
*O*. *glazioviana*	57.59±4.33a	281±41a	269±36a
*E*. *haichowensis*	46.49±4.11b	456±68b	429±57b

Mean value followed by different letter is statistically significant (ANOVA; Duncan multiple range test, p<0.05).

#### Structural characteristics of the leaf epidermis

**Fig 6:**
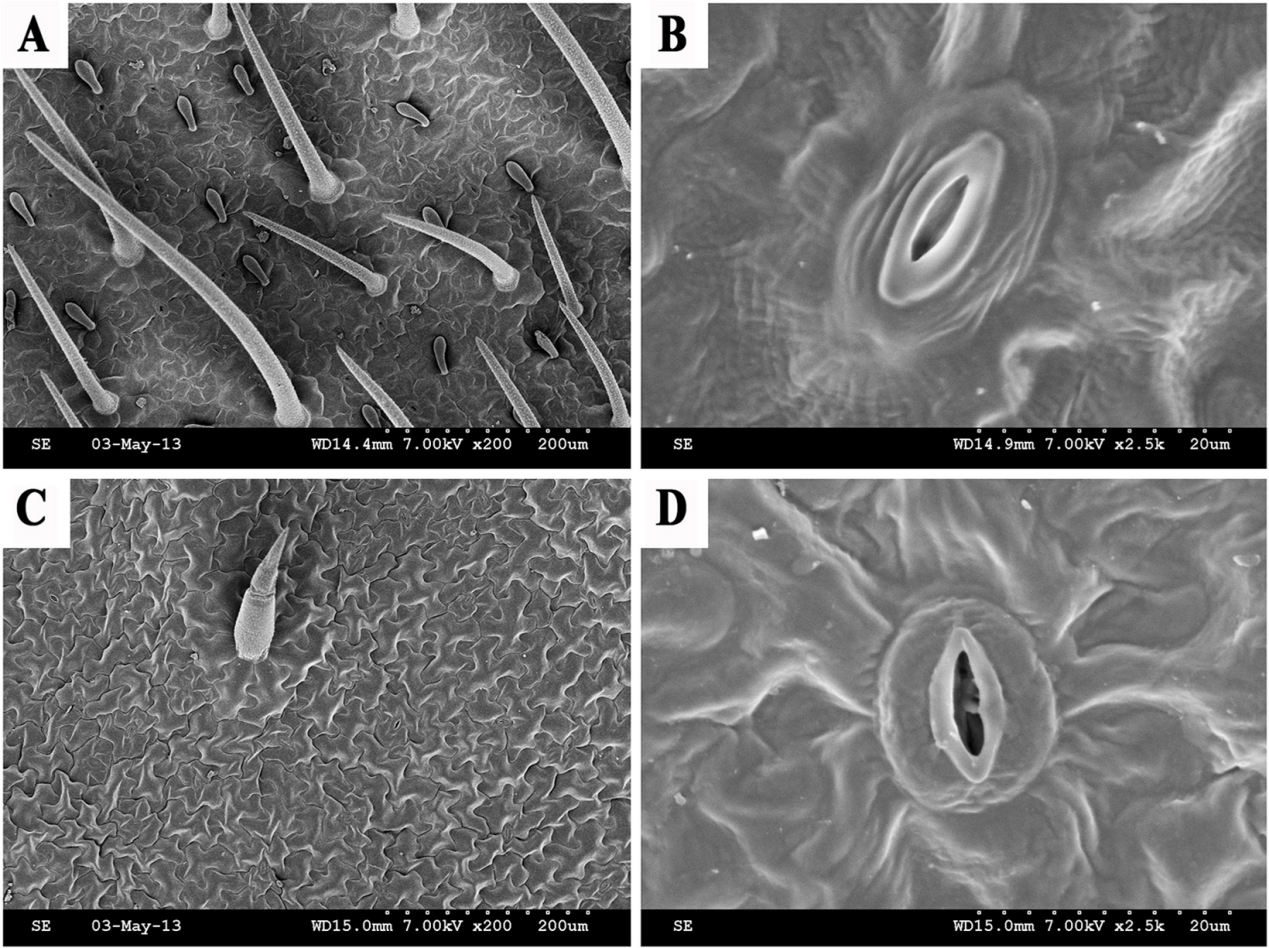
The lower epidermis as shown by SEM. (**A**) Lower epidermis of *O*. *glazioviana*, (**B**) magnifying epidermal stoma of *O*. *glazioviana*, (**C**) lower epidermis of *E*. *haichowensis*, and (**D**) magnifying epidermal stoma of *E*. *haichowensis*.

SEM was used to view the characteristics of the hypodermis (Fig 6). The leaf surface of *O*. *glazioviana* was covered with regular, close-knit epidermal hair and many granular pieces of wax, and wax was smoothly distributed at the edge of the stomatal apparatus. By contrast, only a small amount of wax was present near the sparse epidermal hair on the leaf surface of *E*. *haichowensis*. Furthermore, the epidermal surface was rough, with many sags. Less wax was distributed on epidermal cells, but the wax was relatively clear. The greater amount of wax distributed on *O*. *glazioviana* leaves affected the efficiency of transpiration in this species.

The epidermal cells of *O*. *glazioviana* were rugged and tightly packed, and stomata and epidermal hair were scarce. The epidermal cells of *E*. *haichowensis* also had irregular shapes but were more spread out than those of *O*. *glazioviana*. Moreover, the quantities of stomata and epidermal hair on *E*. *haichowensis* leaves were greater compared with *O*. *glazioviana* and had a star-like distribution. The epidermal hair of *O*. *glazioviana* was more numerous and longer compared with *E*. *haichowensis*, shielded most stomata and formed a relatively independent system for reducing water transpiration. The epidermal hair of *E*. *haichowensis* was relatively thin and short and provided the stomata with a relatively open system. The stomata of both plants sank slightly into the epidermis and had guard cells without accessory cells, and ordinary epidermal cells were found irregularly around the stomata. The stomatal guard cells of *O*. *glazioviana* were covered with a thick cuticle layer and formed lip-like shapes; this feature can reduce water evaporation.

#### Microscopic structure of plant root cross sections

**Fig 7:**
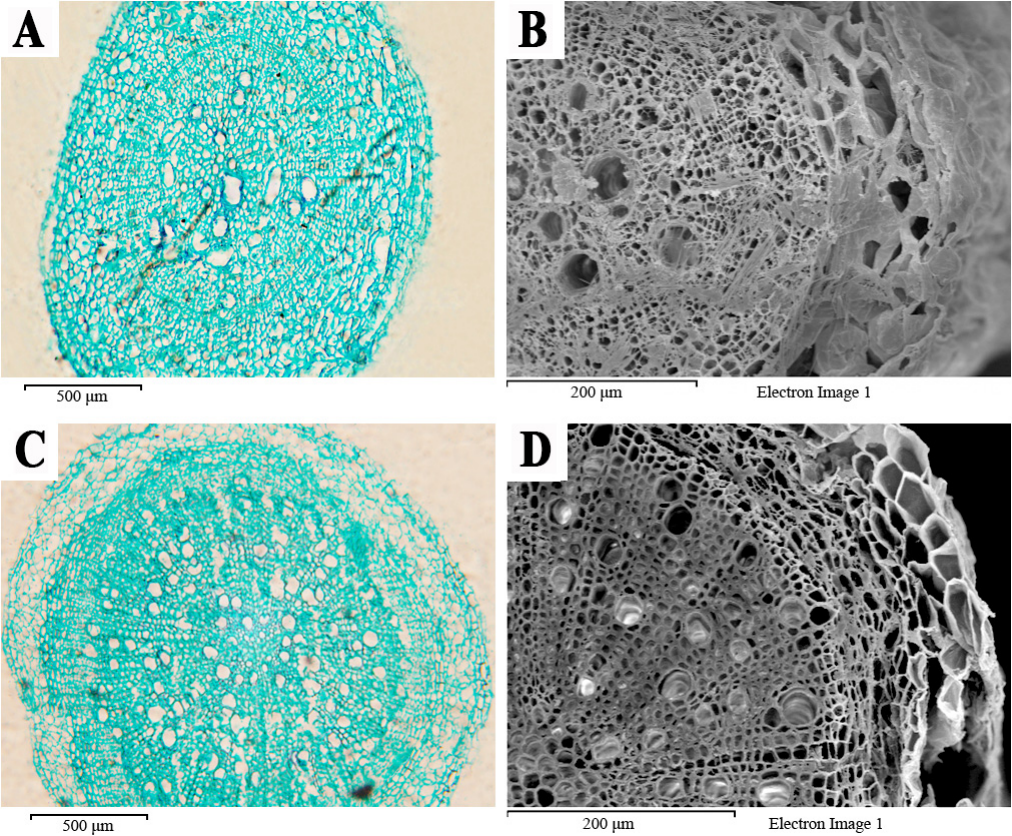
Microstructure of the root cells of two plant species. (**A**) Paraffin cross section of *O*. *glazioviana*, (**B**) electron micrograph of *O*. *glazioviana* cross-section, (**C**) paraffin cross section of *E*. *haichowensis*, and (**D**) electron micrograph of a cross section of *E*. *haichowensis*.

Fig 7 correspond to the root microstructures of *O*. *glazioviana* and *E*. *haichowensis*, respectively. The roots of both plants comprise the epidermis, cortex and stele.

The epidermis of *O*. *glazioviana* was thicker, with neatly arranged cells and mild cutinisation. The outer cortex cells were arranged loosely, and intercellular space was apparent between the cells. The radial and transverse cell walls of endodermal cells exhibited a lignified Casparian strip, which blocks the apoplast pathway between the cortex and stele. The centre of the *O*. *glazioviana* stele did not have pith and primarily played roles in conduction and support. In the xylem, the differentiation of vessels was weak; a small number of loosely arranged vessels was present, and the vessel diameter was irregular. These structures extended the horizontal distance of water transport against the upward transport of water.

The epidermal cells of *E*. *haichowensis* were tightly arranged. The cell walls did not cutinise and were thus suitable for the passage of water and solutes. The outer cortex cells were large and tightly organised, without visible intercellular space, and the endodermal cells were small. The stele of *E*. *haichowensis* accounted for a large proportion of the entire root cross section, with a myelinated centre and obvious differentiation of the primary and secondary xylem. The primary xylem in the cross section was radial, and the vessel aperture primarily comprised numerous regular circles. The primary xylem gradually matured from the outside inward to shorten the transverse distance over which water was conducted, thereby improving the rate of conduction. The vascular cambium split to form rays, and the vessels in the secondary xylem were numerous. The column proportion, the degree of stele differentiation and the many vessels in *E*. *haichowensis* should greatly improve the absorbance and transport of water and mineral elements in the root system. SEM images and paraffin tissue section analyses showed that the stele of *O*. *glazioviana* is small, with fewer loosely arranged vessels. This structure substantially affected the horizontal and vertical transport of water and inorganic ions.

## Discussion

Cu accumulation in plants occurs in several steps, including root absorption, radial transportation, xylem loading and transportation from the root to the shoot. Heavy metals in the roots primarily accumulate in the vacuoles and apoplasts, particularly in the cell wall [28,34]. For several Ni-, Zn-, Cu-, and Pb-tolerant plants, the cell wall plays a key role in preventing excessive quantities of free heavy metals from accumulating in the cytoplasm [35,36,37]. In addition, Cu uptake is also affected by ploidy and ecotypes in the same species[38,39]. Both species of plants examined in the present study can tolerate high Cu concentrations.

Variance analysis of Cu adsorption and accumulation in the root cell walls of both plants indicated that *O*. *glazioviana* cell walls had significantly higher Cu accumulation and adsorption rates compared with *E*. *haichowensis* and that the time to equilibrium for the dynamic adsorption process was significantly longer in *O*. *glazioviana* than in *E*. *haichowensis*. Hence, we inferred that the cell walls of *O*. *glazioviana* roots prevent additional Cu accumulation in the cells and that fewer negatively charged cationic binding sites are present in the cell walls, thus resulting in higher copper exclusion when treated with high Cu concentrations.

Long-distance transportation in plants is an important topic of research in plant physiological ecology. This transportation is involved in the plant water balance, the regulation of stomata, photosynthesis and adaption to different environments [40]. Transpiration plays a primary role in these processes. We found that the Tr of *O*. *glazioviana* was significantly lower than that of *E*. *haichowensis*, particularly when treated with 50 μM Cu. Whether the upward transportation of Cu in *O*. *glazioviana* plays a role in its weak transpiration remains unknown. We used different PEG concentrations to reduce the flow of water in *O*. *glazioviana* to restrain its transpiration and found a significant positive correlation between the Tr and Cu accumulation in plant shoots.

ABA, which is a half terpene compound, was originally considered to be a growth inhibitor that can promote stomatal closure, thereby affecting transpiration [41]. In the present study, the endogenous ABA levels between the roots and the shoots of *O*. *glazioviana* and *E*. *haichowensis* did not differ even when treated with 50 μM Cu. However, exogenous ABA treatment significantly reduced the Tr and the Cu contents in the shoots of both plants. These studies indicate that transpiration plays an important role in Cu accumulation in plant shoots. Previous studies have reported relationships between transpiration and the transport of heavy metals, including Se, Cu, Cd and Pb. Salah and Barrington believed that transpiration promotes the transport of Cd and water through apoplasts [42]. In *Brassica juncea*, Cd is transported to shoots with the flow of transpiration, and exogenous ABA significantly inhibits the accumulation of Cd in shoots[43]. ABA pretreatment inhibits the long-distance, upper transportation of Pb[44]. Buckwheat and wheat exhibited different absorption values for Cu and Zn with different Tr [45].

Plant transpiration is composed of lenticular transpiration, cuticular transpiration and stomatal transpiration, with stomatal transpiration dominating these processes [46]. The density of stomata can affect stomatal transpiration [47]. In the present study, the *O*. *glazioviana* leaf hypodermis was found to have a low density of stomata and long epidermal hair, which both help to reduce excessive transpiration. In addition, plant cuticular wax is a protective screen that stops moisture or solutes in plant epidermal cells from diffusing outward [48]. Thus, the content and thickness of cuticular wax is thought to influence cuticular transpiration [49]. The *O*. *glazioviana* leaf has high surface wax content, which may be one of the reasons why *O*. *glazioviana* has a low Tr.

The xylem vessel comprises a string of highly specialised vascular cells that have perforations where they join each other, and every cell is called a vessel section [50]. Xylem vessels primarily conduct moisture and inorganic salts in plants [51]. When metal ions penetrate root cortical cells, some ions are transported into nearby vacuoles, and other ions penetrate the xylem and then shoots. After the Casparian strip forms in the root endodermis, metal ions cannot directly reach the xylem vessel through the apoplast pathway and must be conducted by the symplast pathway, by xylem loading, upward transportation and accumulation via transpiration [52]. The stele of *O*. *glazioviana* is small, and the vessels are arranged loosely. These structures greatly affect the horizontal and vertical transport of moisture and inorganic ions.

Compared with *E*. *haichowensis*, the root cell wall of *O*. *glazioviana* exhibits weaker Cu adsorption and accumulation and a slower adsorption rate. Leaf SEM images and root tissue section analysis indicated that the Tr of *O*. *glazioviana* was significantly lower than that of *E*. *haichowensis* due to the higher leaf wax content, greater quantity of epidermal hair, smaller numbers of stroma stoma, and smaller xylem vessel cross-sectional areas present in *O*. *glazioviana*. This lower Tr makes Cu ion entry into the root organisation of *O*. *glazioviana* and the Cu ion transport process from the bottom to top of *O*. *glazioviana* plants more difficult. We inferred that the differences in the structural characteristics between these two plants are the primary factors affecting the low extent to which Cu is accumulated and absorbed in *O*. *glazioviana*.

## Conclusion

The study is focused in diverse physiological responses and structural characteristics between *O*. *glazioviana* (Cu-exclusion type) and *E*. *haichowensis* (Cu-enrichment type) related to the Cu uptake from a hydroponic solution. Supported by an important number of experimental variables, the result can be of interest for the characterization of plant species oriented to be used in phytoremediation programs. However, further investigation of Cu uptake, translocation, and storage in *O*. *glazioviana* and *E*. *haichowensis* using genetic and molecular techniques will be required to achieve an understanding of the mechanisms.
